# Associations between classic psychedelics and opioid use disorder in a nationally-representative U.S. adult sample

**DOI:** 10.1038/s41598-022-08085-4

**Published:** 2022-04-07

**Authors:** Grant Jones, Jocelyn A. Ricard, Joshua Lipson, Matthew K. Nock

**Affiliations:** 1 Department of Psychology, Harvard University, 33 Kirkland St, Cambridge, MA 02138 USA; 2 Yale University, New Haven, USA; 3 Teachers College, Columbia University, New York, USA; 4 Harvard University, Cambridge, USA

**Keywords:** Psychology, Human behaviour

## Abstract

Opioid use disorder (OUD) is a major source of morbidity and mortality in the U.S. and there is a pressing need to identify additional treatments for the disorder. Classic psychedelics (psilocybin, peyote, mescaline, LSD) have been linked to the alleviation of various substance use disorders and may hold promise as potential treatments for OUD. The aim of this study was to assess whether the aforementioned classic psychedelic substances conferred lowered odds of OUD. Furthermore, this study aimed to replicate and extend findings from Pisano et al. (2017) who found classic psychedelic use to be linked to lowered odds of OUD in a nationally representative sample. We used recent data from the National Survey on Drug Use and Health (2015–2019) (*N* = 214,505) and multivariable logistic regression to test whether lifetime use (yes/no) of classic psychedelics was associated with lowered odds of OUD. Lifetime psilocybin use was associated with lowered odds of OUD (aOR: 0.70; 95% CI [0.60, 0.83]). No other substances, including other classic psychedelics, were associated with lowered odds of OUD. Additionally, sensitivity analyses revealed psilocybin use to be associated with lowered odds of seven of the 11 DSM-IV criteria for OUD (aOR range: 0.66–0.83). Future clinical trials and longitudinal studies are needed to determine whether these associations are causal.

## Introduction

Opioid use disorder (OUD) is a major public health crisis, with nearly 70,000 opioid-related deaths in the United States in 2017 alone, representing a 292% increase since 2001^1,2^. Opioids, including heroin, fentanyl, and prescription opioid compounds, are responsible for approximately 70% of all overdose deaths in the United States^3^. Additionally, over the course of the COVID-19 pandemic, mortality related to opioid overdoses has reached alarming new highs^4^, with the largest increases among members of racial and ethnic minority groups, as well as in areas of socioeconomic disadvantage^5^. Two gold-standard pharmacological treatments exist for opioid dependence—methadone and buprenorphine, both of which are synthetic opiate derivatives. Unfortunately, these common interventions come with high risk of dependence, therefore often trading one addiction for another. There is a clear need to identify more effective interventions for OUD as well as to explore protective factors that may increase the likelihood of abstinence from these addictive compounds^6–8^.

Classic psychedelics (Greek for “mind-manifesting”) represent potential treatments for OUD; furthermore, studying these substances can potentially help to identify protective factors for OUD as well. Classic psychedelics are a group of psychoactive compounds found in nature or synthesized from natural precursors. Such compounds often produce mystical-type or ego-dissolution experiences in users^9^. The major compounds included in this class of substances include: lysergic acid diethylamide (LSD), psilocybin, ayahuasca, dimethyltryptamine (DMT), peyote, and mescaline. Following a several-decade hiatus*,* recent years have seen a promising revival of research on the use of psychedelics in the treatment of addiction*.* A strong body of preliminary work has emerged to support psychedelic-assisted therapy for substance dependence and abuse, with studies supporting the efficacy for psilocybin as a smoking cessation treatment*,* as well as LSD as a treatment for alcoholism^10–12^. Additionally, in a sample of patients who self-administered psychedelics (e.g., LSD and psilocybin) in a naturalistic context and reported the effects of psychedelics before and after administration, 96% met substance use disorder (SUD) criteria before psychedelic use, whereas following psychedelic use, only 27% met criteria for a SUD^13^. These findings offer preliminary support for classic psychedelics as a treatment for addiction.

As limited as the literature on the relationship between psychedelics and addiction is, the body of research examining the associations between classic psychedelic use and OUD is even smaller. A few studies have hinted that the association is worth exploring, however. In an online study, Garcia-Romeu et al. found a decrease in opioid and cannabis cravings after naturalistic experiences with classic psychedelics^13^. Recently, Argento and colleagues^14^ analyzed a longitudinal cohort of 3813 people who use drugs and found naturalistic psychedelic use to predict lowered odds of daily opioid use. Additionally, Pisano et al. conducted a landmark study in this line of inquiry, examining the association between the use of classic psychedelics and incidence of OUD within a large population sample of illicit drug users. Using data from the National Survey of Drug Use and Health (NSDUH) (2008–2013), Pisano et al. demonstrated that classic psychedelic use conferred 27% reduced risk of past-year opioid dependence and 40% reduced risk of past-year opioid abuse^15^.

Given the overall dearth of research into the association between psychedelic use and opioid dependence in a naturalistic context, it is crucial to examine whether such findings replicate. It is also necessary to understand the unique associations that individual classic psychedelics share with OUD. Work from Jones and Nock^16–19^ found that different psychedelic compounds have differing patterns of association with mental health outcomes. Thus, examining the correlates between lifetime use of several commonly-used psychedelic compounds and OUD can help to identify the specific compounds best suited to treating OUD.

This paper uses the latest NSDUH data to replicate and extend the findings from Pisano et al. on the associations between naturalistic use of psychedelics and OUD, as well as to examine the associations between use of particular classic psychedelic substances (psilocybin, peyote, mescaline, and LSD) and OUD. To our knowledge, this study represents the first attempt since Pisano et al. to investigate this association in the context of a large, nationally representative sample.

## Method

Data are from The National Survey on Drug Use and Health (NSDUH) (2015–2019), an annual survey that examines substance use and health outcomes within a nationally-representative sample of the United States population. Individuals experiencing homelessness, active-duty military members, and currently incarcerated citizens are not surveyed by the NSDUH. We included all adults aged ≥ 18 years from the NSDUH in our analyses (total unweighted *N* = 214,505). This study was exempt from IRB review as NSDUH data are publicly available and all methods in this study were carried out in accordance with relevant guidelines and regulations.

### Analyses

We used a similar analytical approach to Pisano et al. We used the ‘Survey’ package in R to conduct survey-weighted multivariable logistic regression models that incorporated the complex survey design and weighting included in the NSDUH^20^. Our main model tested the association between classic psychedelics and past year diagnosis of OUD (dependence or abuse). For any classic psychedelics associated with lowered odds of OUD, we conducted sensitivity analyses to assess the associations between these substances and each of the 11 *DSM-IV* criteria for opioid dependence and abuse^21^. This analytical approach utilizes virtually all OUD-related variables in the NSDUH and allows for detailed insight into the associations between classic psychedelics and OUD.

### Independent variables and covariates

Our main independent variables were lifetime use (yes/no) of the four most frequently used classic psychedelics in the NSDUH: psilocybin, peyote, mescaline, and LSD. We also included the following demographic factors and substances as a priori covariates within our analyses: sex, age, race/ethnicity, educational attainment, self-reported engagement in risky behavior, annual household income, marital status, and lifetime use of various substances (MDMA/ecstasy, PCP, inhalants, cocaine, tranquilizers, stimulants, sedatives, and marijuana).

### Dependent variables

The main dependent variable was past year diagnosis of OUD. Individuals met criteria for OUD if they abused or experienced dependence on heroin or prescription pain relievers in the past year. Additionally, each of the 11 *DSM-IV* diagnostic criteria for opioid dependence or abuse served as dependent variables in our sensitivity analyses. The criteria were as follows:

1. Spent a great deal of time over a period of a month getting, using, or getting over the effects of opioids.
2. Unable to keep or set limits on opioid use or used opioids more often than intended.
3. Needed to use opioids more than before to get desired effects or noticed that using the same amount had less effect than before.
4. Unable to cut down or stop using opioids every time he/she/they tried or wanted to.
5. Continued to use opioids even though they were causing problems with emotions, nerves, mental health, or physical health.
6. Reduced or gave up participation in important activities due to opioid use.
7. Reported experiencing three or more opioid withdrawal symptoms at the same time that lasted longer than a day after opioid use was cut back or stopped (i.e. feeling blue, nausea/vomiting, fever, etc.).
8. Serious problems at home, work, or school caused by using opioids.
9. Used opioids regularly and then did something that might have put the user in physical danger.
10. Use of opioids caused someone to do things that repeatedly got him/her/them in trouble with the law.
11. Problems with family or friends caused by using opioids and continued to use opioids even though the user thought opioids caused these problems.

For our sensitivity analyses, all aforementioned demographics and lifetime use variables served as covariates.

## Results

### Demographics

Differences in the demographic characteristics of those with and without past year OUD are presented in Table 1. Participants with past year OUD are more likely to be single, less formally educated, younger, male, white, lower income, and report more frequent engagement in risky behavior.

**Table 1:** Demographics of those with versus without opioid use disorder (OUD).

Characteristic	Does not have OUD (weighted %) (N = 212,322)	Has OUD (weighted %) (N = 2183)	p-value^1^
**Marital status**			< 0.001
Married	51.9	27.6	
Widowed	5.9	3.1	
Divorced or separated	13.8	19.8	
Never been married	28.4	49.5	
**Education**			< 0.001
5th grade or lower	1.2	0.5	
6th grade	1.1	0.4	
7th grade	0.5	0.6	
8th grade	1.1	1.5	
9th grade	1.8	2.9	
10th grade	2.1	3.9	
11th or 12th grade	5.0	8.2	
High school diploma/GED	24.8	32.5	
Some college credit	21.5	26.8	
Associate's	9.3	8.4	
Bachelor's or higher	31.7	14.4	
**Age**			< 0.001
18–25	13.9	18.4	
26–34	15.9	27.5	
35–49	24.6	28.6	
50 +	45.6	25.5	
**Sex**			< 0.001
Male	48.2	58.4	
Female	51.8	41.6	
**Race**			< 0.001
Non-Hispanic White	63.8	72.7	
Non-Hispanic Black	11.9	10.4	
Non-Hispanic Native American/Alaska Native	0.5	0.8	
Non-Hispanic Native Hawaiian/Pacific Islander	0.4	0.2	
Non-Hispanic Asian	5.6	1.6	
Non-Hispanic more than one race	1.7	2.9	
Hispanic	16.1	11.3	
**Yearly household income**			< 0.001
< $20,000	16.2	31.2	
$20,000–$49,999	29.4	31.8	
$50,000–$74,999	16.0	14.0	
$75,000 +	38.4	23.0	
**Self-reported engagement in risky behavior**			< 0.001
Never	55.6	27.6	
Seldom	32.0	35.6	
Sometimes	11.2	31.0	
Always	1.3	5.8	

^1^Chi-squared test with Rao and Scott's second-order correction.

### Associations between psilocybin and OUD

The results from our main model testing the associations between lifetime use of classic psychedelics (psilocybin, peyote, mescaline, LSD) and past year OUD are presented in Table 2. Psilocybin was the only substance associated with lowered odds of OUD (aOR: 0.70; 95% CI [0.60, 0.83]). All other substances examined, including other classic psychedelics (peyote, mescaline, and LSD), shared no association with OUD or were associated with increased odds of OUD.

**Table 2:** Associations between lifetime use of various substances and opioid use disorder (OUD).

Lifetime use	Frequency (unweighted N)	aOR (95% CI)^1^
Psilocybin	22,276	0.70*** (0.60, 0.83)
Peyote	3766	0.84 (0.63, 1.12)
Mescaline	4595	1.13 (0.86, 1.49)
LSD	22,552	1.15 (0.94, 1.42)
MDMA/ecstasy	21,195	1.66*** (1.35, 2.03)
PCP	3935	1.63** (1.25, 2.12)
Cocaine	32,783	3.54*** (2.89, 4.34)
Inhalants	21,856	1.44*** (1.21, 1.73)
Tranquilizers	48,572	3.40*** (2.79, 4.14)
Stimulants	32,033	1.44*** (1.24, 1.68)
Sedatives	27,218	1.93*** (1.61, 2.30)
Marijuana	110,175	2.39*** (1.71, 3.35)

^1^*p < 0.05; **p < 0.01; ***p < 0.001; *aOR* adjusted odds ratio, *CI* confidence interval. Significant values that indicate lowered odds of OUD are in bold.

The results of our sensitivity analyses testing the associations between lifetime psilocybin use and past year presence of each of the 11 *DSM-IV* criteria for opioid dependence and abuse are presented in Table 3. Psilocybin use was associated with lowered odds of seven of the 11 criteria for opioid dependence and abuse (aOR range: 0.66–0.83). Additionally, psilocybin was marginally associated with lowered odds of two additional *DSM-IV* criteria for opioid dependence and abuse as well (aOR range: 0.75–0.80; *p* < 0.10).

**Table 3:** Associations between psilocybin and the 11 DSM-IV criteria for opioid dependence and abuse.

Opioid dependence and abuse criteria	Frequency (unweighted N)	aOR (95% CI) (psilocybin as independent variable)^1^
1. Significant time spent getting/using	2249	0.83* (0.70, 0.98)
2. Use more than intended	1030	0.71* (0.54, 0.93)
3. Decreased effects/need more for same effect	2901	0.82* (0.70, 0.96)
4. Unable to cut back	914	0.80^†^ (0.62, 1.04)
5. Emotional/physical health problems	1383	0.73** (0.60, 0.90)
6. Fewer important activities	1320	0.71** (0.58, 0.87)
7. 3 + Withdrawal symptoms	1724	0.86 (0.71, 1.05)
8. Significant work/home/school problems	1063	0.66** (0.50, 0.86)
9. Use in physically hazardous situations	910	0.66** (0.49, 0.88)
10. Recurrent legal trouble	454	0.72 (0.45, 1.15)
11. Social/interpersonal issues	906	0.75^†^ (0.56, 1.01)

^1†^p < 0.10; *p < 0.05; **p < 0.01; ***p < 0.001; *aOR* adjusted odds ratio, *CI* confidence interval. Significant values that indicate lowered odds of OUD criteria are in bold.

### Post-hoc analyses of psilocybin users who have versus have not misused opioids

In addition to our sensitivity analyses, we also conducted post-hoc chi-squared analyses to assess whether there are significant demographic differences between psilocybin users who have versus have not ever misused opioids (i.e., tried heroin and/or used prescription pain relievers to get high). Differences between these two groups would suggest that third-variable demographic variables partially mediate the associations between psilocybin and lowered odds of OUD, as this finding would suggest that there exists a unique population of psilocybin users that has never misused opioids and subsequently are at little to no risk for OUD. These findings are reported in Table 4. Our post-hoc analyses revealed that psilocybin users who have versus have not used opioids differed from one another on all of the demographic traits assessed: marital status, education level, age, sex, race/ethnicity, and yearly household income.

**Table 4:** Demographic differences for psilocybin users who have versus have not misused opioids.

Characteristic	Lifetime psilocybin use only (weighted %) (N = 11,992)	Lifetime psilocybin use plus opioid misuse (weighted %) (N = 10,284)	p-value^1^
**Marital status**			< 0.001
Married	47.2	37.3	
Widowed	2.4	2.1	
Divorced or separated	17.0	18.0	
Never been married	33.4	42.7	
**Education**			< 0.001
5th grade or lower	0.2	0.1	
6th grade	< 0.1	< 0.1	
7th grade	0.1	0.2	
8th grade	0.4	0.8	
9th grade	0.7	1.1	
10th grade	1.4	1.9	
11th or 12th grade	3.1	4.4	
High school diploma/GED	19.2	23.6	
Some college credit	24.9	28.9	
Associate's	10.9	9.4	
Bachelor's or higher	39.2	29.5	
**Age**			< 0.001
18–25	11.9	13.5	
26–34	17.9	27.2	
35–49	29.3	30.4	
50 +	40.9	28.9	
**Sex**			< 0.001
Male	64.0	68.6	
Female	36.0	31.4	
**Race**			< 0.001
Non-Hispanic White	82.3	84.6	
Non-Hispanic Black	2.5	1.9	
Non-Hispanic Native American/Alaska Native	0.5	0.6	
Non-Hispanic Native Hawaiian/Pacific Islander	0.2	0.2	
Non-Hispanic Asian	2.3	1.3	
Non-Hispanic more than one race	2.6	2.8	
Hispanic	9.5	8.4	
**Yearly household income**			< 0.001
< $20,000	13.4	16.4	
$20,000–$49,999	25.1	29.5	
$50,000–$74,999	15.9	16.3	
$75,000 +	45.6	37.9	

^1^Chi-squared test with Rao and Scott's second-order correction.

## Discussion

Psilocybin was the sole classic psychedelic substance associated with lowered odds of past year OUD in a large, nationally-representative sample of the U.S. population. These findings accord with other population-based survey research indicating that classic psychedelics share differing relationships to mental health outcomes in naturalistic contexts^16–19^. Additionally, the magnitude of the association between psilocybin use and OUD (30% reduction in odds) is comparable to that initially reported by Pisano et al. using NSDUH data from 2008 to 2013—allowing us to report that Pisano et al.’s findings replicate in a different (more recent) nationally-representative sample. The association between lifetime use of psilocybin and OUD was not driven by a few particular criteria for OUD; rather, lifetime psilocybin use was significantly associated with reduced odds of seven out of 11 *DSM-IV* criteria for opioid dependence and abuse.

These results are cross-sectional and correlational and so cannot be used to make causal inferences. However, this study offers an important contribution to the research literature by demonstrating the replication of Pisano et al.’s original finding that lifetime use of psychedelics conferred lowered odds of opioid dependence and abuse. As clinically minded researchers pursue trials aimed at demonstrating the therapeutic efficacy of psilocybin-based treatments for opioid addiction, our study provides a foundation for this line of inquiry with preliminary evidence from a naturalistic context. Furthermore, our findings suggest it is worth investigating the protective effects of psilocybin for all related diagnostic criteria for OUD, including overuse and tolerance, opioid-related emotional distress, and opioid-related social and work problems.

### Limitations

These results should be interpreted in the context of several important limitations. Above all, given that our results are based on cross-sectional data, they cannot be used to draw causal conclusions. Casual investigations (e.g., clinical trials) are needed to better understand the nature of the association between psilocybin use and lowered odds of OUD. Additionally, all of the questions we drew from the NSDUH are based on self-report. As a result, for questions on both psychedelic use and OUD, under-reporting might be a confound in our analyses and conclusions.

Next, although we controlled for many demographic covariates, there are many we likely could not control for due to the limitations of the NSDUH dataset. For instance, the NSDUH does not assess information about homelessness status nor collect information from individuals who are currently incarcerated or serving as active-duty military members. Not accounting for demographic factors such as these may lead to an overestimation of the strength of the association between psilocybin use and lowered odds of OUD. Future research should attempt to control for these factors to maximize the integrity of any observed relationships between psilocybin use and OUD.

Finally, items assessing psychedelic use asked about “lifetime” use, but not recency or frequency, precluding examination of these features of psychedelic use. However, given that psilocybin has been shown to elicit lasting reductions in substance dependence after just two to three administrations, causal interpretations of our findings remain plausible even in light of this limitation^22^.

### Potential mediators

A number of potential mediators might underlie the current study’s findings. First, as mentioned in Pisano et al., the effects of psilocybin on the serotonin system might mediate its protective association with OUD. Classic psychedelic compounds, including psilocybin, act primarily as serotonin (5-HT_2A_) agonists, meaning that they bind to a receptor site typically targeted by serotonin^23^. Abnormal serotonin neurotransmission is linked to many aspects of addiction, such as craving and heightened responses to drug cues^24–26^. Furthermore, there is suggestive evidence that serotonin agonists may support the treatment of opioid addiction as they may indirectly inhibit the release of dopamine^27^, a key neurotransmitter that is implicated in the maladaptive reward system changes associated with opioid addiction^28^. At present, pharmacological explanations of our findings remain purely speculative. Future research on the pharmacology of psilocybin can potentially shed further light on the link between this compound and lowered odds of OUD.

Second, the mystical-type experiences induced by psilocybin represent another key mechanism that might explain that protective associations between psilocybin and OUD. There is suggestive evidence of this mediation effect within clinical explorations of psychedelics for the treatment of addiction. In Johnson et al.'s open-label trial of psilocybin for nicotine dependence, improvement was correlated with measures of mystical experience and spiritual significance of psilocybin sessions^29^. More broadly, spiritual experience and belief have been linked to positive substance abuse recovery outcomes^30–32^. Further inquiry into the role of mystical-type experiences in promoting recovery from addiction can help to elucidate the nature of the observed link between psilocybin use and reduced odds of OUD.

Lastly, third-variable pre-drug factors may plausibly underlie the protective associations observed within our study as well. Prior research has demonstrated there are pre-drug differences associated with psychedelic users (e.g. greater extroversion, higher levels of spirituality) that may simultaneously confer lowered odds of adverse mental health outcomes such as OUD^33–37^.

Additionally, demographic differences associated with psilocybin use and OUD may contribute to our observed associations. Our post-hoc analyses revealed significant demographic differences between psilocybin users who have versus have not misused opioids on all of the demographic dimensions we assessed (marital status, education level, age, sex, race/ethnicity, and yearly household income). Although we accounted for these demographic variables in our analyses, as previously stated, there are possibly additional demographic factors that we could not control for that mediate our findings. Future research should more closely investigate how demographic differences impact the associations between psilocybin use and lowered odds of OUD. These investigations may have the additional benefit of identifying protective factors associated with the alleviation of OUD. Lastly, if these inquires reveal demographic differences associated with the use of specific psychedelic compounds (i.e. differing populations that use psilocybin versus peyote), these inquiries may also clarify why psilocybin—and no other classic psychedelic substance—conferred lowered odds of OUD.

## Conclusion

This study replicates Pisano et al.’s finding linking classic psychedelics to lowered odds of opioid use disorder across a broad spectrum of diagnostic criteria, but specifies that this link only exists for psilocybin, and not for LSD or phenethylamine psychedelics (mescaline and peyote). Future clinical trials will be required to test whether this association is causal, and to identify which mediators may underlie this association. Longitudinal studies should also assess whether there is a causal link between psilocybin and OUD. These studies can also provide essential foundational evidence for the link between psilocybin and OUD and maximize the likelihood of ethical and safe clinical trials that assess the treatment potential of this compound. In conclusion, our study represents an incremental step towards a greater understanding of factors that may prevent or alleviate opioid use disorder.
